# Annotations

**Published:** 1898-03-12

**Authors:** 


### Annotations.

### Judges and Abusive Language.

It is high time that the public learned that the *ex cathedra* utterances of judges, coroners, magistrates, and such-like people, in regard to matters which are not specially brought before them by evidence which can be cross-examined, are of no special importance or authority. Nothing can more impress one with the necessity of submitting all evidence to the test of cross-examination than the remarks which even judges are led to make when they are tempted, as, unfortunately, they only too often are, to comment on matters which come informally before them. In a case which was recently tried at the Lambeth County Court, the solicitors to the defendants appear to have asked one of the resident surgeons at Guy's Hospital to furnish them with a report as to the condition of the plaintiff when she entered that institution, and this he appears to have sent, asking at the same time for his fee of two guineas. On this letter being put in, Judge Emden is reported to have said: "For a responsible professional man in a hospital to exact these high fees from the poor is shameful. It is a miserable piece of extortion, of which this is not the first instance which has come under my notice." All of this, of course, is nonsense. As was explained afterwards in a letter to the Press, the "report" spoken of was not for the poor plaintiff, but for the rich defendant. It is perfectly clear that the surgeon was under no obligation whatever to write it, and that if the solicitors to the defendants wanted the evidence they could have obtained it by subpœna. If, however, they wished to have the satisfaction of a report on a purely professional topic, they ought to be prepared to pay professional fees; and as for "extortion," a county-court judge, of all people, ought to know that the price of a thing is what it will fetch. The report was not necessary for the bringing of the matter to justice; the evidence was perfectly accessible by the ordinary process of law; the report was, in fact, a mere luxury, which the surgeon was under no obligation whatever to provide; and for the judge to speak of the asking of a fee for such a document as an "extortion" was to use an expression which was by no means warranted by the facts of the case, and would, if only for the sake of the dignity of his office, have been much better left unsaid.

### The Advantages of Professional Reticence.

While we by no means agree with Judge Emden's criticism on the action of the house surgeon at Guy's Hospital, in asking a fee for a report on the case of a patient under his care, we think that this action opens up a question of great professional interest. Is it right to give such reports at all, either with or without a fee? Take the case above referred to (one in which the plaintiff made a claim for injuries sustained in consequence of an alleged defect in the coal-cellar plate on premises occupied by the defendant), what right had the house surgeon to give to the defendant a report on the condition of the plaintiff on admission? We are only speaking in accordance with recognised professional canons when we say that, except in a court of law, and by the direction of the judge, no medical man should reveal to others any facts concerning his patient which he has become cognisant of when acting in a professional capacity. The matter has come up again and again in regard to house surgeons and their relation with the police, complaints having been made that hospital authorities do not give information. It is clear, however, that for a surgeon to make himself an agent of the police would be to break the most binding rules as to professional secrecy. But how are we to maintain these rules, and how are we to persuade people as to the existence even of such a code, if medical men are willing to give reports, such as the one to which we have alluded, to any solicitor who may be willing to offer a good fee? It cannot be too strongly laid down that a medical man's best protection from the annoyances of the Law Courts is to be found in a proper and correct observance of accepted rules as to professional reticence. Criminal cases cannot be helped; but when we find house surgeons appearing in civil cases, we may be pretty sure that it is to a large extent their own fault. A silent tongue will keep at bay even a speculative attorney, for few people will call a witness of the nature of whose evidence they are ignorant. We commend this to the consideration of all medical men who dislike appearing in court. If they maintain the rule of silence until ordered to speak by a magistrate or a judge, they will very seldom be called as witnesses. It is the preliminary "reports" and "certificates" and chatter which do the mischief.

### The Modern Mother.

It is to be feared that one result of the increased employment of women in wage-earning industries, and of the delegation of all educational influences to the paid school teacher who teaches out of books rather than by example, is a gradual degradation of those habits of personal cleanliness, of that particularity and carefulness about all that concerns the body, the hair, and the clothing which even among the poorest distinguishes the well brought up child of the careful mother from the child of the streets. A letter in the New York *Medical Record*, from a medical man in Washington, reveals a condition of affairs which is a sad commentary on that emancipation of woman which we are led to imagine has gone further in America than in other and more benighted portions of the globe. The Medical Inspector of Schools is quoted as having, in January of this year, reported on the prevalence of lousiness, or, as it appears in his report, "pediculosis," in Boston's public schools. In one school there were 756 children, all but 50 of them girls, and mostly from respectable families. Of the whole 756 children only 200, or 26 per cent., were free from vermin. But more unsatisfactory still was the fact that, when they were given a couple of weeks during the Christmas holidays in which to clean themselves up, it was found on their return to school that out of 509 of them whose heads were examined only 26 were free from nits. Such a degree of apathy and indifference on the part of the parents in presence of a condition which would have excited the liveliest feelings of disgust in a mother of the old school speaks ill, indeed, for the home training of both the mothers and the daughters of the present generation.

